# The obesogenic environment around elementary schools: food and beverage marketing to children in two Mexican cities

**DOI:** 10.1186/s12889-018-5374-0

**Published:** 2018-04-07

**Authors:** Simón Barquera, Lucia Hernández-Barrera, Stephen J. Rothenberg, Enrique Cifuentes

**Affiliations:** 1 Nutrition and Health Research Center, National Institute of Public Health, Cuernavaca, Morelos Mexico; 2 Center for Research in Population Health, National Institute of Public Health, Cuernavaca, Morelos Mexico; 3 Department of Environmental Health, Harvard TH Chan School of Public Health, Boston, MA USA

**Keywords:** Food-marketing, Child obesity, Nutrition, Sugar-sweetened beverages

## Abstract

**Background:**

Unhealthy environments and food advertisements are major determinants of childhood obesity. Recent regulation has banned unhealthy foods from schools in Mexico. However, currently there is no regulation limiting exposure to food marketing around schools. Thus, our objective was to analyze the characteristics of food advertising practices around 60 elementary schools in two cities and to evaluate compliance with the Pan American Health Organization (PAHO) recommendations and the local food industry self-regulatory marketing code.

**Methods:**

Data were collected during the period of October 2012 to March 2013. A random sample of elementary schools was selected from two Mexican cities. Using geographic information systems, we drew a 100-m-diameter buffer around each school. Trained personnel obtained photographs to assess the locations and types of food advertisements. Our results were stratified by school type and by indicators of compliance with the PAHO and industry recommendations. We developed a multivariate negative binomial regression model to determine factors predicting the number of advertisements around schools.

**Results:**

The number of advertisements was significantly higher around public schools than around private schools (6.5 ± 5.6 vs. 2.4 ± 3.5, *p* < 0.05). Printed posters were the most common type of marketing medium (97%), showing mostly sugar-sweetened beverages, sweet breads, candies, and bottled water. Promotions, such as special prices or gifts, were included on 30% of printed posters. Food advertising practices were often in compliance with industry recommendations (83%) but not with those from the PAHO (32%) (*p* < 0.001).

**Conclusion:**

Our results support the importance of monitoring the obesogenic environment and identifying policy tools to protect children from food marketing not only inside schools but also around them, particularly in lower income communities.

## Background

Childhood obesity is a major public health problem around the world, in particular in developing countries, where it has shown an alarming increasing trend in the last 30 years [1–4]. Recently, diverse studies have identified exposure to unhealthy environments as a major determinant of obesity in populations experiencing what is termed the nutrition transition [5–8]. These obesogenic environments are typically characterized by low active transportation and poor physical activity, infrastructure, and facilities, together with increased access to low-cost, ready-to-eat, energy-dense foods and sugar-sweetened beverages promoted by ubiquitous marketing strategies [4, 9–11]. One of the most important drivers of this epidemic is a shift in the food system, which is affecting dietary intake. Together, these conditions promote inactivity, sedentary lifestyles, and excessive caloric consumption, resulting in high obesity prevalence [2].

In Mexico, results from the National Health and Nutrition Surveys from 1988 to 2012 show that overweight and obesity prevalence in school-aged children (5–11 years) has increased over the last 20 years. Currently, overweight and obesity affect 34% of all school-aged children in the country [12, 13]. The Pan American Health Organization (PAHO) has published recommendations in relation to food and beverage marketing to children stating that there should be no marketing technique on any communication channel to promote foods high in fats, sugars, or salt to children, including no marketing communications in places where children gather and spend time such as schools and parks. The list of marketing channels includes, among others, outdoor printed advertising, in-store marketing, celebrity endorsements, incentives, and prize promotions. However, the food industry allocates a substantial portion of their marketing budget to influencing children’s consumption preferences [14–16]. Using actors, athletes, artists, celebrities, cartoons, attractive designs and messages, innovative media, discounts, gifts, and promotions, advertisements for many products of poor nutritional value are able to influence children’s choices [17–19].

At the time this study was undertaken, there was no regulation of food and beverage marketing to children in Mexico. In 2009, the Council of Ethical Self-Regulatory Marketing, created by the food industry, established a code of ethics to address marketing of food and beverages directed to children known as PABI (from the Spanish, Publicidad de Alimentos y Bebidas Dirigidas al Público Infantil) [20] as a proactive measure to avoid federal regulation. This code established 30 ethical norms in addition to mechanisms to enable the code’s control and application [21]. This self-regulatory code has been criticized and is considered insufficient to protect children, as becomes apparent when it is compared with international recommendations from health organizations [14, 22]. Some of the most conspicuous differences between these norms and international recommendations from health agencies and expert groups are the following: a) differences in the age groups used to define children < 12 years of age in the self-regulatory code vs. < 16 years; b) self-regulatory code based on industry consensus vs. state regulated; c) monitoring and evaluation by an industry-nominated committee vs. by an independent council; d) self-regulatory code containing ambiguous nutritional profiles that allow some products high in sugar, fat, or sodium to be marketed to children vs. no marketing to children; and e) a self-regulatory code allowing the promotion of products directed to children using characters and other strategies which are not recommended by international health agencies [14, 23, 24].

The environment around schools may contribute in different ways to the children’s nutritional status; i.e. active transportation opportunities and the influence of marketing are two major examples of how this environment affects physical activity or food choices. Furthermore, active transportation promotion intended to prevent obesity, might contribute to child’s weight gain if intensive marketing exposure is present. However, health researchers from developing countries have barely described this phenomenon. In Latin America, studies have been conducted to investigate TV advertising of food and beverages (in Mexico, Colombia, Guatemala, and Brazil, among others) and marketing inside schools [23, 25–28]. Marketing inside schools, a common practice by the major soda and snack brands, was forbidden under the healthy school guidelines introduced in Mexico in 2010 [29–31]. Preliminary evaluations of this policy are promising, but results differ according to socioeconomic status (SES) [27]. Schools in the poorest areas of the country have been less effective in implementing the guidelines, suggesting that special attention must be paid to vulnerable groups to promote healthier and less obesogenic environments [27]. However, advertising around schools is harder to characterize and regulate. Walton et al. evaluated the number of food stores and advertisements that children encounter during the trip from home to school and back in New Zealand, finding that 87% of children were exposed to at least one food advertisement on the house–school–house route [32]. Other studies have suggested that this type of advertising could influence children’s food and beverage choices [17, 33]. Yet, this environmental factor is not considered by current obesity prevention efforts, and school routes could be targeted to reduce unhealthy choices made by children.

The objective of this study was to describe the presence of food and beverages advertising around schools in two cities in Mexico and to evaluate compliance with the PAHO recommendations on marketing of food and non-alcoholic beverages to children and with the Mexican food industry’s self-regulatory marketing code regarding marketing of foods and beverages to children.

## Methods

### Study design and sample selection

This study was carried out in the cities of Cuernavaca and Guadalajara to characterize the obesogenic environment around elementary schools. Data were obtained from October to December 2012 in Cuernavaca and from January to March 2013 in Guadalajara. These periods assured that surveys were collected always during school days. The universe of schools from each city was obtained from the Ministry of Education school registry in Cuernavaca (*n* = 200 of those 99 were private and 101 public schools) and Guadalajara (*n* = 714 of those 562 were private and 152 public schools). Stratified probabilistic sampling techniques were used. Random selection was done using Epidata, version 3.1. Strata were represented by the schools’ categories (i.e., public or private), and all schools had the same probability of being selected. Type of school (private or public) is considered a proxy of SES, since private schools charge fees that most families from low-income groups cannot afford. We randomly selected approximately 30 schools per city from the total universe using Epidata software [34]. A total of 29 schools in Cuernavaca (private = 16, public = 13) and 31 in Guadalajara (private = 11, public = 20) formed the final sample group (*n* = 60). Maps were generated using the main door of selected schools as centers of computer-generated circular buffers with a 100-m diameter. The size of the buffer was defined based on the minimum recommended proximity criteria that should be free from food and beverage advertising, according to the PAHO recommendations and on budget constraints [14]. From the 100-m diameter circular area, we assessed the non-school section of the circle. We adjusted our results for the buffer size, depending on the size of the school’s facilities.

These buffer zones were inspected for convenience stores, markets, cafeterias, restaurants, and any other kind of commercial source of food and beverage advertising. The owners of the establishments provided informed consent to conduct this investigation. The Institutional Review Board from the National Institute of Public Health approved the study in April 2012. Additional details on the methodology have been published elsewhere [35].

### Data collection

We conducted an inventory of advertisements showing industrial food products, processed food and beverages (except alcohol) inside and outside the convenience stores. Advertisements were defined as any poster, banner, sticker, painting on walls, or flags inside or outside stores, and billboards and walls not related to the stores, in the buffer areas.

Trained and standardized field personnel collected data and photographs of the food advertisements. We used a format that allowed us to capture the characteristics of the food product, i.e., brand name, type of advertisement, and use of promotions. We followed quality control procedures for coding of advertisements, data georeferencing, and classification. Using this procedure, we were able to quantify the total number of convenience stores surrounding schools and of advertisements inside and outside stores. Direct audits were conducted during school hours (Monday-Friday 9:00 am to 5:00 pm). The data were classified into the following categories: soda, juices, and sugar-sweetened beverages; sweet snacks (e.g., donuts and desserts); chocolates and candies; milk and dairy products; salty/fried snacks; water; ice cream, and other foods. Finally, we evaluated their compliance with the PAHO recommendations for marketing to children and the Mexican food industry’s self-regulatory PABI code.

### Compliance with the PAHO recommendations for marketing to children

We evaluated whether advertisements around the school buffer zones were in compliance with three selected PAHO criteria (Table 1): use of characters, location; promotions, incentives and discounts. We also evaluated whether this marketing complied with three selected dimensions of the food industry’s PABI self-regulatory code; price, characteristics and status (Table 1). Although there are other criteria in both the recommendations and the code, those listed here are the ones relevant to the environment around elementary schools.

**Table 1:** PAHO and self-regulatory PABI code criteria definitions used to identify food industry compliance in marketing

PAHO	
2003Selected criteria	Definition
Use of characters	Not using or related to animated characters, cartoons, actors, musicians, artists or sports celebrities, and children.
Location	In recommendation 7, the PAHO expert consultation group established that “marketing” should be defined as all marketing techniques through all communication channels, including messages disseminated in schools and other places where children gather and spend time. These places include schools, parks, sport centers, nurseries, doctors’ offices, and any other places where children congregate. In terms of this study, we defined any advertisement within 100 m of a school as marketing in places where children gather and spend time.
Promotions, incentives, and discounts	The PAHO recommends restricting advertisements for foods high in saturated fat, trans fatty acids, sugar free, or salt (including special offers and incentives such as sweepstakes, coupons, or other discounts).
PABI code (Industry pledge)	
Selected criteria	Definition
Price	The price of the food must be declared in a concrete and understandable manner. Use of specific words such as “only” and “less than” should be avoided when referring to price.
Characteristics	According to PABI, publicity has to be accurate when showing food or beverage product characteristics, without assigning nutritional values or superior characteristics to those that the product has. It is important to mention that the code does not include specific criteria to evaluate food product characteristics. Therefore, we considered advertisements as non-compliant with the code when the images shown did not correspond with product characteristics such as flavor, size, content, and nutritional properties. The most common case of non-compliance we found was that of nutritional values in advertisements showing images of food (fruits) that a product (soda) does not contain.
Status	It is forbidden to mislead or confuse a child with the idea of a characteristic of superiority that would be acquired by consuming the product, such as increased strength, popularity, or attractiveness.

### Marginalization index

As a proxy for socioeconomic development, we used the National Population Council 2010 marginalization index. This index applies a score per basic sampling unit calculated using principal component analysis from data obtained from the National Population and Household Census 2010, considering four dimensions: access to health care, access to education, characteristics of the household, and availability of basic goods.

### Statistical analysis

Descriptive statistics were calculated, stratified by public and private schools, and indicators of compliance with PAHO marketing recommendations and the self-regulatory PABI code were compared. We used a chi-square test to compare categorical variables in different schools’ categories (i.e., public or private, used here as proxy of SES); we used Poisson regression models to compare the number of stores, and we also used a negative binomial regression model for the number of advertisements observed in the surroundings of those schools. Then we used a two-sample test of proportions to compare the school’s category in terms of the characteristics of those advertisements. A multivariate analysis of the main factors predicting the number of advertisements around schools was estimated. A multivariate logistic regression model was calculated to analyze the probability of compliance of a food advertisement depending on environmental and community factors. To determine factors predicting the number of advertisements around schools, we developed a negative binomial regression model to account for schools with no convenience stores or food businesses around them, and thus no advertisements. We selected counting methods based on the differences between observed and estimated probabilities for each counting, different likelihood tests and information criteria concerning alternative counting models [36]. All statistical analyses were conducted using the statistical software Stata, version 13.0 [37]. The ethics, biosecurity, and research commissions at the National Institute of Public Health authorized all procedures related to this research.

## Results

We found 103 convenience stores and mini-markets around 43 of the participating schools in the two cities and these establishments were more frequently observed surrounding public schools than private ones. In total, we observed 278 food and beverage advertisements around the schools. The number of advertisements in convenience stores was significantly higher around public schools than around private schools (6.5 ± 5.6 vs. 2.4 ± 3.5, *p* < 0.05) (Table 2). A total of 30.2% of the food advertisements were located inside the stores, while most were located outside (69.8%). Only three billboards marketing alcoholic beverages were registered in the buffer areas of both cities, thus this information was excluded from the analysis.

**Table 2:** Main sample characteristics

		Type of School		
		Public	Private	Total
		n (%)	n (%)	n (%)
		33 (55)	27 (45)	60 (100)
City	Cuernavaca	13 (39.4)	16 (59.3)	29 (48.3)
	Guadalajara	20 (60.6)	11 (40.7)	31 (51.7)
Marginalization score for secondary sampling units	Medium	11 (33.3)	5 (18.5)	16 (26.7)
	Low	15 (45.5)	13 (48.1)	28 (26.7)
	Very Low	7 (21.2)	9 (33.3)	16 (46.6)
Compliance with recommendations	WHO/PAHO	62 (30.5)	22 (36.1)	84 (32)
	PABI	166 (81.8)	53 (86.9)	219 (83)
Advertisements	Inside store	71 (33.3)	13 (20.0)	84 (30.2)
	Outside store	142 (66.7)	52 (80.0)	194 (69.8)
		Mean (SD)	Mean (SD)	Mean (SD)
Convenience stores		1.9 (1.3)	1.0 (1.4) *	1.5 (1.4)
Advertisements		6.5 (5.9)	2.4 (3.5) *	4.6 (5.4)
Advertisements per 100 m^2^ (median P25–75)		0.25 (0.10–0.51)	0 (0–0.27) **	0.13 (0–0.41)

* Statistically significant differences between public and private schools, p < 0.01 using Poisson regression for convenience stores and negative binomial regression for advertisements

** *p* < 0.05 using Median test

Of the total number of advertisements collected (*n* = 278), 50.7% were for sugar-sweetened beverages—mainly soda and juices—followed by sweet breads and snacks (15.8%) and candy and chocolates (9.4%). A small number of bottled water advertisements were also observed (3.2%). We observed similar percentages of food products around both public and private schools. Only in the case of milk and dairy products did we find a higher proportion of advertisements around public schools than around private schools (8.9% vs. 3.1%, *p* < 0.050) (Table 3). Of the total number of advertisements, 29.9% had some type of promotion: special price (18.4%), gifts (7.9%), discounts (1.1%), or another type of promotion (2.5%). The most common medium used was printed posters (97.1%).

**Table 3:** Characterization of marketing around elementary schools

	Type of School		
	Public	Private	Total
Food group	N (%)	N (%)	N (%)
Soda and juice	108 (50.7)	33 (50.8)	141 (50.7)
Bakery and sweet snacks	33 (15.5)	11 (16.9)	44 (15.8)
Chocolate and candy	20 (9.4)	6 (9.2)	26 (9.4)
Milk and dairy products	19 (8.9)	2 (3.1)	21 (7.6)
Fried/salty snacks	11 (5.2)	5 (7.7)	16 (5.8)
Water	6 (2.8)	3 (4.6)	9 (3.2)
Ice cream	3 (1.4)	2 (3.1)	5 (1.8)
Other	13 (6.1)	3 (4.6)	16 (5.8)
Promotion type			
None	146 (68.5)	49 (75.4)	195 (70.1)
Special offer	43 (20.2)	8 (12.3)	51 (18.4)
Discount	1 (0.5)	2 (3.1)	3 (1.1)
Gift	17 (8.0)	5 (7.7)	22 (7.9)
Other	6 (2.8)	1 (1.5)	7 (2.5)
Marketing type			
Printed advertisements (e.g., posters and brochures)	208 (97.7)	62 (95.4)	270 (97.1)
Illuminated advertisements	3 (1.4)	–	3 (1.2)
Animated advertisements	2 (0.9)	3 (4.6)	5 (1.8)
Total	213 (100)	65 (100)	278 (100)

No significant differences were found using two-sample test of proportions

We found significant differences in the compliance of the marketing of food and beverage products with the PAHO recommendations and with the self-regulatory PABI code. The percentage of advertisements complying with the WHO/PAHO recommendations was smaller than that complying with the PABI code (32% vs. 83%; *p* < 0.01) (Table 2). The lowest compliance was observed in the PAHO recommendation concerning the use of famous characters in food advertisements (71.2%), followed by promotion messages (70.8%) (Data not shown in tables). The logistic regression model showed that the probability of complying with the PAHO criteria was higher for advertisements complying with the PABI code (OR = 1.13, *p* = 0.07) and lower in Guadalajara than in Cuernavaca (OR = 0.81, p = < 0.01). The probability of complying with the PAHO criteria was also lower when the product advertised was milk and dairy products (OR = 0.64, *p* = 0.010) (Table 4).

**Table 4:** Probability of compliance with the PAHO recommendations by associated factors (n = 278)

Factor	Odds ratio (95% CI)	*p*-value
Compliance with PABI code		
No	1	
Yes	1.13 (0.99–1.30)	0.07
School type		
Private	1	
Public	0.95 (0.85–1.06)	0.37
City		
Cuernavaca	1	
Guadalajara	0.81 (0.74–0.89)*	< 0.01
Marginalization score by basic geostatistical area		
Medium	1	
Low	1.10 (0.98–1.23)	0.11
Very low	1.11 (0.98–1.27)	0.10
Food Marketing		
Soda and juice	1	
Bakery and sweet snacks	0.95 (0.83–1.08)	0.42
Chocolate and candy	0.85 (0.71–1.00)	0.06
Milk and dairy products	0.64 (0.46–0.90)*	0.01
Fried/salty snacks	1.02 (0.84–1.23)	0.84
Water	0.93 (0.72–1.19)	0.55
Ice cream, sherbets, and popsicles	1.44 (1.00–2.07)	0.05
Other	0.86 (0.69–1.07)	0.17

*Statistically significant differences, *p* < 0.05

When we assessed potential factors associated with the frequency of food and beverage advertisements around schools using negative binomial regression, we found that public schools had significantly (*p* < 0.001) more advertisements in stores than private schools (Table 5).

**Table 5:** Negative binomial regression for predictors of the number of advertisements around elementary schools (n = 60)

Factor	Relative risk (95% CI)	p-value
School type		
Private	1	–
Public	2.49 (1.19–5.19)	0.015*
City		
Cuernavaca	1	–
Guadalajara	1.81 (0.88–3.73)	0.104
Marginalization score by basic geostatistical area tertile		
Medium	1	
Low	0.91 (0.39–2.15)	0.836
Very low	0.80 (0.31–2.09)	0.655

*Statistically significant, p < 0.05

## Discussion

The contribution of the environment to the rise in obesity has been extensively described in recent years in diverse parts of the world [2, 7, 38]. To understand and tackle this complex problem, a multidisciplinary and multisectoral approach is necessary, including monitoring and regulation of food industry marketing actions oriented toward influencing children’s preferences [17, 39–42]. Previous studies have documented a substantial presence of marketing to children around schools, and this has been recognized as a contributing factor in child obesity [32, 43]. Our study showed that most elementary schools (*n* = 43) were surrounded by convenience stores that work as food and beverage marketing units. Currently, although there are guidelines being used, there is no enforcement of regulations of these practices. Most marketing materials are designed for placement outside of stores (69.8%), increasing the potential influence and overall impact on the local community. The present study also showed that children from public schools are more likely to be exposed to this kind of marketing than children from private schools, which are typically more affluent and located in wealthier and more protected neighborhoods. A number of studies have found an important relation between food environments, quality of food, food stores and products based on socio-economic characteristics [3]. In our study, children from low-income families were more exposed to a higher presence of marketing and have more access to unhealthy foods than the better-off children from these cities (Cuernavaca and Guadalajara) did. As expected, sugar-sweetened beverages and sweet snacks comprised a major component of these advertisements (66.5%), and as observed in studies from other countries [25, 26, 28, 43–45]. In Mexico, this is a public health concern of major relevance, since Mexico is the highest per capita soda consumer in the world, showing a high prevalence of childhood obesity and diabetes as the leading cause of mortality [12, 46, 47]. A number of policies aimed at reducing consumption of sugar-sweetened beverages by children in particular and by the population in general have been developed recently, such as the healthy hydration recommendations, the national agreement for healthy nutrition, the guidelines for healthy nutrition inside schools, and the soda tax [12, 48, 49]. However, our study documents the need for additional actions to effectively reduce sugar-sweetened beverage and unhealthy food consumption by children.

Only 18.1% of the advertisements complied with the PAHO recommendations for food and beverage marketing to children, as opposed to 83.5% that were in compliance with the food industry’s self-regulatory code, similar to the findings of studies in other countries [21, 26, 43, 50–56]. In addition, the industry self-regulatory code is vague in terms of the parameterization of the evaluated areas, which makes evaluation difficult and subject to interpretation by the evaluation group. This reflects the need to identify mechanisms to enforce regulation based on national and international recommendations from health organizations, which protect children with a health-in-all policies approach and avoid leaving this responsibility to individuals or actors involved in the public health sector alone [57–59]. Of great concern is that 16.5% of the products did not even comply with the food industry’s self-regulatory code, regardless of its laxness in protecting children. The fact that the number of advertisements is associated with the density of convenience stores reflects the important role that these small community food stores play as an open channel contributing to and influencing the exposure of children to food and beverage marketing.

A number of scholars have described the environmental and socioeconomic factors associated with food intake and choices [11, 60]. The nutrition transition observed in Mexico over the last years has modified negatively food choices with some differences by SES. For example, of low income populations consume a greater proportion of less-healthy beverages [61]. Active commuting to school in Mexico is negatively associated with obesity and is currently seen as an activity that may result in health benefits and therefore needs to be supported [62]. On the other hand, marketing of unhealthy foods around schools might reduce some of these benefits, since children are more susceptible to be influenced by marketing [11].

Our study represents a first approximation of the characterization of this phenomenon, but these results must be considered with caution. Although the study is representative of two urban cities in Mexico, its external validity is not assured, and further investigation is needed to understand the magnitude and characteristics of this phenomenon nationwide, particularly in a more heterogeneous range of socioeconomic conditions given the current results pointing to higher marketing intensity in vulnerable, less affluent groups. Our buffer size was small compared to those used in other similar studies, which might account for the lower density of food marketing described here. We selected this buffer size because the required data was not available from any GIS system or database. Thus, we implemented labor-intensive direct observation of field personnel work [25, 32, 43, 53]. We think this conservative approach was sufficient to document the problem with the available resources. Information was obtained from a single visit, so our results did not evaluate seasonality. Thus, some promotions such as those around Children’s Day, sports tournaments, and other events were not necessarily captured. In addition, we did not consider size and other in-depth content of the advertisements or other types of businesses that could potentially sell or market unhealthy foods or beverages. We collected information on non-alcoholic beverages and food; however, the marketing of alcoholic beverages and cigarettes is also a problem that needs to be considered and limited by regulations to protect child health [52]. Finally, this study does not address the impact of advertisements on children’s perceptions or knowledge about nutrition choices or the effects on diet; evidence of this association has been extensively discussed elsewhere [11, 63, 64].

A substantial number of policies that contribute to controlling the obesity epidemic have recently been enacted in Mexico. These include a soda and sugar-sweetened beverage tax, a new food-labeling system, and new guidelines for healthy nutrition in schools [12]. One important challenge is to monitor and evaluate the impact of these policies. Another major challenge is to identify complementary actions that are needed to make these policies effective. In this case, the new school regulations and guidelines might be at risk of having only a modest effect if conditions around schools remain unfavorable or, even worse, if marketing around schools increases and becomes endemic in response to tighter regulations inside schools and through other media channels. The fact that most marketing around schools originates in food businesses and is mostly located outside the stores might imply that relatively simple local regulations could contribute to improving the environment without the need for federal legislation. However, without proper regulation, companies that market to children will be able to redirect their efforts to alternative strategies such as discounts or to different media channels (i.e., from schools and TV to billboards, sports, and/or the Internet) [65–70]. Thus, comprehensive legislation to protect children from food advertisements around schools and in other contexts is crucial. This is an area where action is urgent and necessary, given not only the impact on children but also the documented targeting of vulnerable populations with the potential of generating important health disparities [21, 71, 72]. Exploring the characteristics of an obesogenic environment could be an effective approach to identifying other similarly important areas of opportunity to complement obesity prevention actions—specifically, how to increase physical activity, improve access to nutrition information, and disincentivize misleading marketing practices [59, 72].

## Conclusions

Our results support the importance of monitoring the obesogenic environment and identifying ways to protect children from food marketing not only inside elementary schools but also around them, particularly children from public schools and low-income neighborhoods who may be more exposed to food marketing than children from private schools and better-off neighborhoods. As in other public health policies, planning should consider potential unintended effects and design mechanisms to prevent them. In this particular case, a coordinated local action aligned to the federal policies is necessary to avoid marketing strategies aimed at maintaining consumption of unhealthy foods by children at schools.

## Abbreviations

PABI: (from the Spanish) Publicidad de Alimentos y Bebidas Dirigidas al Público Infantil
PAHO: Pan American Health Organization
SES: Socio Economic Status

## References

[1] Ng M, Fleming T, Robinson M, Thomson B, Graetz N, Margono C, Mullany EC, Biryukov S, Abbafati C, Abera SF (2014). Global, regional, and national prevalence of overweight and obesity in children and adults during 1980-2013: a systematic analysis for the global burden of disease study 2013. Lancet.

[2] Popkin BM, Slining MM (2013). New dynamics in global obesity facing low- and middle-income countries. Obes Rev.

[3] Monteiro CA, Moura EC, Conde WL, Popkin BM (2004). Socioeconomic status and obesity in adult populations of developing countries: a review. Bull World Health Organ.

[4] Popkin BM (2007). Understanding global nutrition dynamics as a step towards controlling cancer incidence. Nat Rev Cancer.

[5] Swinburn B, Vandevijvere S, Kraak V, Sacks G, Snowdon W, Hawkes C, Barquera S, Friel S, Kelly B, Kumanyika S (2013). Monitoring and benchmarking government policies and actions to improve the healthiness of food environments: a proposed government healthy food environment policy index. Obes Rev.

[6] Potvin Kent M, Dubois L, Wanless A (2012). A nutritional comparison of foods and beverages marketed to children in two advertising policy environments. Obesity (Silver Spring).

[7] Mackenbach JD, Rutter H, Compernolle S, Glonti K, Oppert J-M, Charreire H, De Bourdeaudhuij I, Brug J, Nijpels G, Lakerveld J (2014). Obesogenic environments: a systematic review of the association between the physical environment and adult weight status, the SPOTLIGHT project. BMC Public Health.

[8] Adeigbe RT, Baldwin S, Gallion K, Grier S, Ramirez AG (2015). Food and beverage marketing to Latinos: a systematic literature review. Health Educ Behav.

[9] James WPT (2008). The fundamental drivers of the obesity epidemic. Obes Rev.

[10] Monteiro CA, Moubarac JC, Cannon G, Ng SW, Popkin B (2013). Ultra-processed products are becoming dominant in the global food system. Obes Rev.

[11] Larson N, Story M (2009). A review of environmental influences on food choices. Ann Behav Med.

[12] Barquera S, Campos I, Rivera JA (2013). Mexico attempts to tackle obesity: the process, results, push backs and future challenges. Obes Rev.

[13] Barquera S, Campos-Nonato I, Hernandez-Barrera L, Pedroza A, Rivera-Dommarco JA (2013). Prevalence of obesity in Mexican adults 2000-2012. Salud Publica Mex.

[14] Pan American Health Organization (PAHO). Recommendations from a Pan American Health Organization Expert Consultation on the Marketing of Food and Non-Alcoholic Beverages to Children in the Americas. In*.* Washington, DC.:; 2011: 130.

[15] Federal Trade Commission. Marketing Food to Children and Adolescents: A Review of Industry Expenditures, Activities, and Self-Regulation. Washington, DC: U.S. Federal Trade Commission; 2008.

[16] Federal Trade Commission. A review of food marketing to children and adolescents: Follow-up report. Washington, DC: U.S. Federal Trade Commission; 2012.

[17] Kelly B, King L, Baur L, Rayner M, Lobstein T, Monteiro C, Macmullan J, Mohan S, Barquera S, Friel S (2013). Monitoring food and non-alcoholic beverage promotions to children. Obes Rev.

[18] Kraak VI, Story M (2015). An accountability evaluation for the industry's responsible use of brand mascots and licensed media characters to market a healthy diet to American children. Obes Rev.

[19] IOM (Institute of Medicine). Challenges and Opportunities for Change in Food Marketing to Children and Youth: Workshop Summary. Washington, DC: The National Academies Press; 2013. 24872975

[20] Consejo de Autorregulación y Ética Publicitaria. Código PABI. Código de Autorregulación de Publicidad de Alimentos y Bebidas No Alcohólicas dirigida al Público Infantil. Ciudad de México: CONAR; 2012.

[21] Sharma LL, Teret SP, Brownell KD (2010). The food industry and self-regulation: standards to promote success and to avoid public health failures. Am J Public Health.

[22] World Health Organization. A Framework for Implementing the Set of Recommendations on the Marketing of Foods and Non‐alcoholic Beverages to Children. Geneva: World Health Organization; 2012.

[23] Theodore F, Juarez-Ramirez C, Cahuana-Hurtado L, Blanco I, Tolentino-Mayo L, Bonvecchio A (2014). Barriers and opportunities for the regulation of food and beverage advertising to children in Mexico. Salud Publica Mex.

[24] Bragg MA, Yanamadala S, Roberto CA, Harris JL, Brownell KD (2013). Athlete endorsements in food marketing. Pediatrics.

[25] Chacon V, Letona P, Barnoya J (2013). Child-oriented marketing techniques in snack food packages in Guatemala. BMC Public Health.

[26] Chacon V, Letona P, Villamor E, Barnoya J (2015). Snack food advertising in stores around public schools in Guatemala. Crit Public Health.

[27] Barquera STM, Safdie M, National L (2014). Guidelines for healthy nutrition in Mexican schools: an independent preliminary evaluation. Obes Rev.

[28] Mallarino C, Gomez LF, Gonzalez-Zapata L, Cadena Y, Parra DC (2013). Advertising of ultra-processed foods and beverages: children as a vulnerable population. Rev Saude Publica.

[29] Hernández Ávila M, Martínez Montañez OG. Lineamientos generales para el expendio o distribución de alimentos y bebidas en los establecimientos de consumo escolar en los planteles de educación básica. Boletín médico del Hospital Infantil de México. 2011;68:1–6.

[30] DOF. ACUERDO mediante el cual se establecen los lineamientos generales para el expendio o distribución de alimentos y bebidas en los establecimientos de consumo escolar de los planteles de educación básica. 2010. Available from: http://dof.gob.mx/nota_detalle_popup.php?codigo=5156173. Accessed 28 Mar 2018.

[31] Secretaria de Educacion Publica. Lineamientos Generales para el expendio o distribución de alimentos y bebidas en los establecimientos de consumo escolar de los planteles de educación básica. Acuerdo Nacional para la Salud Alimentaria. Estrategia contra el Sobrepeso y la Obesidad: Ciudad de México; 2010.

[32] Walton M, Pearce J, Day P (2009). Examining the interaction between food outlets and outdoor food advertisements with primary school food environments. Health Place.

[33] Cairns G, Angus K, Hastings G, Caraher M (2013). Systematic reviews of the evidence on the nature, extent and effects of food marketing to children. A retrospective summary. Appetite.

[34] Christiansen TB and Lauritsen JM. (Ed.) EpiData - Comprehensive Data Management and Basic Statistical Analysis System. Odense Denmark, EpiData Association; 2010. http://www.epidata.dk.

[35] Barrera LH, Rothenberg SJ, Barquera S, Cifuentes E (2016). The toxic food environment around elementary schools and childhood obesity in Mexican cities. Am J Prev Med.

[36] Long JS, Predicted JF (2001). Probabilities for count models. Stata J.

[37] StataCorp. Stata Statistical Software: Release 13. College Station, TX: StataCorp LP; 2013.

[38] Williams J, Scarborough P, Matthews A, Cowburn G, Foster C, Roberts N, Rayner M (2014). A systematic review of the influence of the retail food environment around schools on obesity-related outcomes. Obes Rev.

[39] Vandevijvere S, Tseng M (2013). Towards comprehensive global monitoring of food environments and policies to reduce diet-related non-communicable diseases. Public Health Nutr.

[40] Popkin BM, Bellagio Meeting g (2013). Bellagio declaration 2013: countering big Food's undermining of healthy food policies. Obes Rev.

[41] Gosliner W, Madsen KA (2007). Marketing foods and beverages: why licensed commercial characters should not be used to sell healthy products to children. Pediatrics.

[42] Harris JL, Graff SK (2012). Protecting young people from junk food advertising: implications of psychological research for first amendment law. Am J Public Health.

[43] Kelly B, Cretikos M, Rogers K, King L (2008). The commercial food landscape: outdoor food advertising around primary schools in Australia. Aust N Z J Public Health.

[44] Mazur A, Telega G, Kotowicz A, Malek H, Jarochowicz S, Gierczak B, Mazurkiewicz M, Pop T, Zajkiewicz K, Druzbicki M (2008). Impact of food advertising on food purchases by students in primary and secondary schools in South-Eastern Poland. Public Health Nutr.

[45] Ng SH, Kelly B, Se CH, Chinna K, Sameeha MJ, Krishnasamy S, Mn I, Karupaiah T (2014). Obesogenic television food advertising to children in Malaysia: sociocultural variations. Glob Health Action.

[46] Stern D, Piernas C, Barquera S, Rivera JA, Popkin BM (2014). Caloric beverages were major sources of energy among children and adults in Mexico, 1999-2012. J Nutr.

[47] Barquera S, Hernandez-Barrera L, Tolentino ML, Espinosa J, Ng SW, Rivera JA, Popkin BM (2008). Energy intake from beverages is increasing among Mexican adolescents and adults. J Nutr.

[48] Secretaría de Salud. Bases técnicas del Acuerdo Nacional para la Salud Alimentaria. Estrategia contra el sobrepeso y la obesidad. Ciudad de México: Secretaría de Salud; 2010.

[49] Rivera JA, Munoz-Hernandez O, Rosas-Peralta M, Aguilar-Salinas CA, Popkin BM, Willett WC, Comite de Expertos: Beverage consumption for a healthy life: recommendations for the Mexican population. Consumo de bebidas para una vida saludable: recomendaciones para la poblacion mexicana. Salud publica de Mexico. 2008;50(2):173–95. 10.1590/s0036-36342008000200011 18372998

[50] Sacks G, Mialon M, Vandevijvere S, Trevena H, Snowdon W, Crino M, Swinburn B (2014). Comparison of food industry policies and commitments on marketing to children and product (re)formulation in Australia, New Zealand and Fiji. Clinical Public Health.

[51] Amanzadeh B, Sokal-Gutierrez K, Barker JC (2015). An interpretive study of food, snack and beverage advertisements in rural and urban El Salvador. BMC Public Health.

[52] Hillier A, Cole BL, Smith TE, Yancey AK, Williams JD, Grier SA, McCarthy WJ (2009). Clustering of unhealthy outdoor advertisements around child-serving institutions: a comparison of three cities. Health Place.

[53] Maher A, Wilson N, Signal L (2005). Advertising and availability of 'obesogenic' foods around New Zealand secondary schools: a pilot study. N Z Med J.

[54] Snowdon W (2014). Sugar-sweetened beverages in Pacific Island countries and territories: problems and solutions?. Pacific health dialog.

[55] Story M, Nanney MS, Schwartz MB (2009). Schools and obesity prevention: creating school environments and policies to promote healthy eating and physical activity. Milbank Q.

[56] Potvin Kent M, Dubois L, Wanless A (2011). Self-regulation by industry of food marketing is having little impact during children's preferred television. Int J Pediatr Obes.

[57] Swinburn B, Sacks G, Vandevijvere S, Kumanyika S, Lobstein T, Neal B, Barquera S, Friel S, Hawkes C, Kelly B (2013). INFORMAS (international network for food and obesity/non-communicable diseases research, monitoring and action support): overview and key principles. Obes Rev.

[58] Vandevijvere S (2014). Why a global convention to protect and promote healthy diets is timely. Public Health Nutr.

[59] Swinburn B, Sacks G, Lobstein T, Rigby N, Baur LA, Brownell KD, Gill T, Seidell J, Kumanyika S, International Obesity Taskforce Working Group on Marketing to C (2008). The 'Sydney Principles' for reducing the commercial promotion of foods and beverages to children. Public Health Nutr.

[60] Hawkes C, Smith TG, Jewell J, Wardle J, Hammond RA, Friel S, Thow AM, Kain J (2015). Smart food policies for obesity prevention. Lancet.

[61] López-Olmedo N, Popkin BM, Taillie LS (2018). The socioeconomic disparities in intakes and purchases of less-healthy foods and beverages have changed over time in urban Mexico. J Nutr.

[62] Jauregui A, Medina C, Salvo D, Barquera S, Rivera-Dommarco JA (2015). Active commuting to School in Mexican Adolescents: evidence from the Mexican National Nutrition and health survey. J Phys Act Health.

[63] Galbraith-Emami S, Lobstein T (2013). The impact of initiatives to limit the advertising of food and beverage products to children: a systematic review. Obes Rev.

[64] Boyland EJ, Whalen R (2015). Food advertising to children and its effects on diet: review of recent prevalence and impact data. Pediatr Diabetes.

[65] Blakely T, Ni Mhurchu C, Jiang Y, Matoe L, Funaki-Tahifote M, Eyles HC, Foster RH, McKenzie S, Rodgers A (2011). Do effects of price discounts and nutrition education on food purchases vary by ethnicity, income and education? Results from a randomised, controlled trial. J Epidemiol Community Health.

[66] Boelsen-Robinson T, Backholer K, Peeters A (2016). Digital marketing of unhealthy foods to Australian children and adolescents. Health Promot Int.

[67] Carter M-A, Signal L, Edwards R, Hoek J, Maher A (2013). Food, fizzy, and football: promoting unhealthy food and beverages through sport - a New Zealand case study. BMC Public Health.

[68] Freeman B, Kelly B, Baur L, Chapman K, Chapman S, Gill T, King L (2014). Digital junk: food and beverage marketing on Facebook. Am J Public Health.

[69] Jenkin G, Madhvani N, Signal L, Bowers S (2014). A systematic review of persuasive marketing techniques to promote food to children on television. Obes Rev.

[70] Kelly B, Bauman AE, Baur LA (2014). Population estimates of Australian children's exposure to food and beverage sponsorship of sports clubs. J Sci Med Sport.

[71] Lobstein T (2013). Research needs on food marketing to children. Report of the StanMark project. Appetite.

[72] Hawkes C (2007). Regulating and litigating in the public interest: regulating food marketing to young people worldwide: trends and policy drivers. Am J Public Health.

